# Unhealthy Dietary Habits and Obesity: The Major Risk Factors Beyond Non-Communicable Diseases in the Eastern Mediterranean Region

**DOI:** 10.3389/fnut.2022.817808

**Published:** 2022-03-16

**Authors:** Ayoub Al-Jawaldeh, Marwa M. S. Abbass

**Affiliations:** ^1^World Health Organization (WHO), Regional Office for the Eastern Mediterranean (EMRO), Cairo, Egypt; ^2^Oral Biology Department, Faculty of Dentistry, Cairo University, Cairo, Egypt

**Keywords:** NCDs, unhealthy diet, obesity, risk factors, EMR

## Abstract

There are 22 countries in the Eastern Mediterranean Region (EMR) expanding from Morocco in the west to Pakistan and Afghanistan in the east, containing a population of 725,721 million in 2020. In the previous 30 years, the illness burden in the EMR has transmitted from communicable diseases to non-communicable diseases such as diabetes, cardiovascular diseases, and cancer. In 2019, cardiovascular mortality in the EMR was mostly attributed to ischemic heart disease, the first reason for mortality in 19 countries in the region. Stroke was the second reason for death in nine countries followed by diabetes, which was ranked as the second reason for death in two countries. The prominent nutrition-related NCDs risk factors in EMR include obesity, hypertension, high fasting plasma glucose, and upregulated unhealthy diet consumption. Most of the EMR population are unaware of their NCDs risk factor status. These risk factors, even if treated, are often poorly controlled, therefore, inhibiting their existence by changing the lifestyle to proper dietary habits and sufficient physical activity is mandatory. In this review, the epidemiology and nutrition-related risk factors of NCDs in the EMR will be discussed and illustrated, aiming to scale up action and support decision-makers in implementing cost effective strategies to address obesity and NCDs prevention and management in the region.

## Introduction

The Eastern Mediterranean Region (EMR) encompasses 22 countries including [Afghanistan, Bahrain, Djibouti, Egypt, Iran, Iraq, Jordan, Kuwait, Lebanon, Libya, Morocco, Oman, Pakistan, Palestine, Qatar, Saudi Arabia, Somalia, Sudan, Syria, Tunisia, United Arab Emirates (UAE), and Yemen], with a population of ~725,720 million (1).

In the past three decades, similar to other developing regions in the world, the EMR has undergone a transmission in the disease burden from primarily communicable disorders, such as lower respiratory infections, to non-communicable diseases (NCDs). NCDs include cardiovascular diseases, cancer, diabetes, and chronic respiratory diseases. In 2012, the rate of death from NCDs in the EMR (654 per 100,000 persons) was higher than the global rate (539 per 100,000 persons) and is expected to peak by 2030. In 2015, nearly 58.4% of total deaths in the EMR were due to NCDs, with the chief cause being CVDs (27.4% of total deaths) (2, 3).

NCDs are the essential global cause of death and are responsible for over 70% of deaths worldwide (3). NCDs were responsible for 41 million of the 57 million fatalities worldwide, 15 million of which were premature (30–70 years). The burden is the greatest among low- and middle-income countries, where 78% of global NCDs fatalities and 85% of premature deaths took place (4). Moreover, globally, NCDs were responsible for 1.62 billion DALYs in 2019, with an increase from 43.2% in 1990 to 63.8% in 2019 (5). In 2019, the number of fatalities in EMR due to CVDs was 1,464,672 million, 431,312 thousand individuals died from cancer, and 186,841 thousand died from diabetes (6).

**Figure 1:**
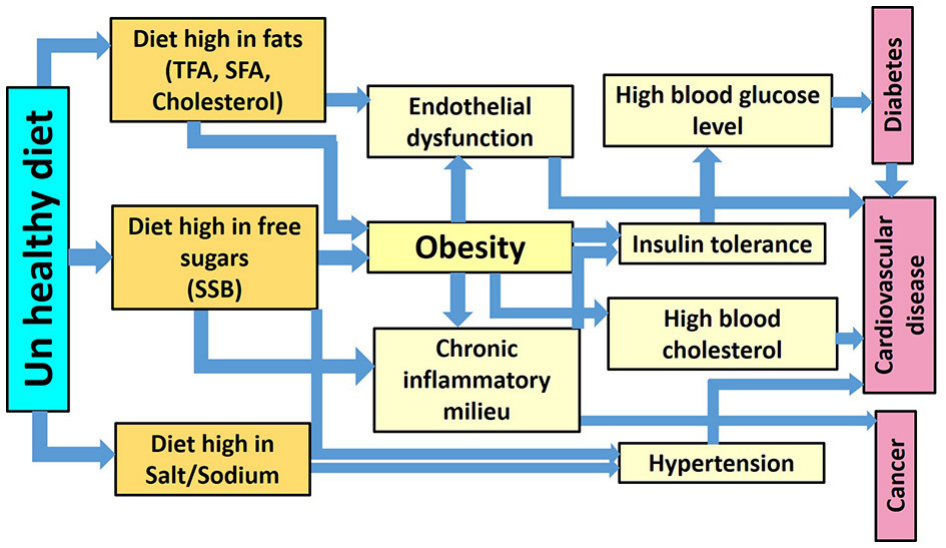
The interdisciplinary relationship between unhealthy diet consumption, obesity, and other NCDs risk factors.

The NCDs share the key four modifiable behavioral risk factors including tobacco usage, unhealthy diet, physical inactivity, and excessive use of alcohol, these factors, in turn, lead to nutritional- physiological related risk factors including overweight/obesity, raised blood pressure, high fasting blood glucose, and high blood cholesterol. The relationship between NCDs and the risk factors involved in their incidence is intermingled and the risk factors are also associated with each other (Figure 1). It is noteworthy that the behavioral risk factors linked with NCDs are closely related to the demographic and socioeconomic status (SES) in the region (7).

Despite NCDs being a critical health obstacle in all EMR countries, tackling NCDs and their key risk factors requires an imperative understanding of the current status and progress at the country and region level. This review discusses nutrition-related NCDs burden and the associated risk factors in the EMR. The current challenges and areas requiring further attention will be also highlighted.

## Methodology

In this paper, the prevalence of NCDs and the associated nutrition-related risk factors in the WHO-EMR are discussed and illustrated. Data for the prevalence of CVDs, diabetes, and cancer as well as different risk factors including overweight/obesity, raised blood pressure, high fasting blood glucose, and high blood cholesterol in the WHO –EMR are summarized. Age-standardized estimates were obtained from the NCDs Risk Collaboration, which in turn, are based on data provided to WHO and the NCDs Risk Factor Collaboration or obtained through a literature review (8). For those estimates, adjustments had been made to standardize risk factor definition, age groups, reporting year, and representativeness of the population. Age-standardized prevalence estimates were calculated to adjust for differences in age/sex structure between populations and to enable comparisons between countries (8). The definition of being overweight or having obesity was used for people with a BMI of 25 kg/m^2^ or higher and a BMI of 30 kg/m^2^ or higher, respectively.

Data regarding the number of deaths and probability of death attributed to CVDs, diabetes, and cancer among adults in EMR were obtained from the global health observatory (9). Raised fasting blood glucose, raised blood pressure, and diabetes prevalence in EMR was also obtained from the global health observatory (6). Cancer trends in EMR data including the number of cancer cases, cancer rates/100,000 (Age-standardized) as well as cumulative cancer risk were obtained from the Global Cancer Observatory (9). Data regarding the food consumption in EMR were collected from the FAO food balance sheets (10). Concerning estimated sodium intakes (g/day) for persons aged 20 and over data were attained from the systematic analysis of 24 h urinary sodium excretion and dietary surveys worldwide (11). Data relating to the mean salt intake for adults (g/day) were obtained from the non-communicable diseases country profiles (4). Saturated fat (% energy), Omega-6 PUFA (% energy), Trans fat (% energy), Dietary cholesterol (mg/d), Seafood omega-3 fat (mg/d), and Plant omega-3 fat (mg/d) data were obtained from the systematic analysis nutrition surveys including 266 countries (11). Data relating to sugar-sweetened beverage consumption in EMR were harvested from a systematic assessment of beverage intake in 187 countries (12).

The data from the Global Burden of Disease Study 2019 presented in this review included the rank of the nutrition related risk factors that caused deaths in EMR countries in 2019 as well as the percentage change in these risk factors between 2009 and 2019 (13).

The policies relating to actions to reduce NCDs in EMR, as well as the policies associated with healthy diets in the countries of the WHO-EMR, are tabulated. Data have been extracted from various sources. These include the WHO's global (6) and regional health observatories (14), data collected for the second WHO Global Nutrition Policy Review 2016–2017 (15), the WHO Global Database on the Implementation of Nutrition Action (GINA) (16), communication about country-level action from WHO country offices and national government nutrition focal points, and other relevant academic papers (17–20). Specifically, data were collected on the policy areas related to a healthy diet that features in the new regional nutrition strategy (21).

Data are presented in narrative or tabular form. To group countries according to the income level, the World Bank classification was used to identify the income level of each country (22). The low-income group includes Afghanistan, Somalia, Sudan, Syria, and Yemen. The lower middle-income group includes Djibouti, Egypt, Morocco, Pakistan, Tunisia, and the occupied Palestinian Territory. The upper middle-income group includes: Iran, Iraq, Jordan, Lebanon, and Libya. The high-income level includes: Bahrain, Kuwait, Oman, Qatar, Saudi Arabia, and United Arab Emirates (UAE).

## Results

### Nutrition Related Non-Communicable Diseases

#### Cardiovascular Diseases

Worldwide, in 2019, cardiovascular diseases were responsible for 393 million DALYs and 18.6 million deaths in both sexes (23). In EMR, the high number of NCDs deaths was attributed to CVDs (1,464,672 million) in 2019. Pakistan recorded the highest number (449,905) followed by Egypt (252,650), Iran (157,018) then Morocco (126,562) (6) (Table 1).

**Table 1:** Number of deaths and probability of death attributed to CVDs, diabetes, and cancer among adults in EMR (6).

	**Number of deaths**			**Number of deaths**			**Number of deaths**			**Probability of dying**			**Probability of**	**Probability of**	**Probability of**
	**attributed to CVDs**			**attributed to diabetes**			**attributed to cancer**			**from any NCDs**			**death due to**	**death due to**	**death due to**
										**between (30–70)**			**CVDs**	**cancer**	**diabetes**
	**Both**	**Males**	**Females**	**Both**	**Males**	**Females**	**Both**	**Males**	**Females**	**Both**	**Males**	**Females**	**Both**	**Both**	**Both**
	**2019**			**2019**			**2019**			**2019**			**2016**		
Afghanistan	71,264	33,793	37,471	8,060	2,612	5,448	15,565	7,756	7,809	35.27	34.37	36.16	21.00	8.00	3.00
Bahrain	1,450	913	537	820	485	336	640	336	303	16.06	16.38	15.43	28.00	16.00	14.00
Djibouti	1,730	913	817	345	196	150	508	206	302	22.01	23.43	20.6	19.00	7.00	2.00
Egypt	252,650	135,587	117,063	26,844	12,863	13,981	85,226	46,452	38,774	28.03	32.74	23.22	40.00	13.00	3.00
Iran	157,018	90,226	66,793	17,947	9,008	8,940	61,063	36,388	24,676	14.80	17.57	11.96	43.00	16.00	4.00
Iraq	62,913	33,304	29,609	9,762	4,731	5,031	15,004	7,594	7,409	23.55	27.52	19.99	27.00	11.00	4.00
Jordan	9,739	4,732	5,007	2,253	1,027	1,227	6,075	3,260	2,815	15.30	17.03	13.57	37.00	12.00	6.00
Kuwait	3,315	2,855	460	321	232	90	1,815	981	835	11.89	13.76	8.03	41.00	15.00	3.00
Lebanon	17,544	10,392	7,151	1,352	783	569	9,078	4,858	4,220	19.87	24.18	15.21	47.00	16.00	5.00
Libya	10,717	5,188	5,530	1,234	514	719	3,557	1,993	1,564	18.59	19.7	17.56	35.00	12.00	4.00
Morocco	126,562	60,732	65,830	10,851	4,346	6,505	33,845	19,293	14,552	24.13	25.97	22.25	38.00	14.00	6.00
Oman	7,848	4,551	3,296	1,143	643	500	1,750	1,131	619	21.48	22.46	20.26	36.00	11.00	8.00
Pakistan	449,905	245,135	204,770	80,976	37,892	43,084	124,328	64,049	60,279	29.41	31.84	26.85	29.00	8.00	3.00
Qatar	1,487	926	561	526	291	235	716	467	248	10.74	10.08	13.24	27.00	16.00	9.00
Saudi Arabia	60,291	38,233	22,058	7,203	4,311	2,892	10,615	6,176	4,438	20.90	22.36	18.44	37.00	10.00	3.00
Somalia	20,301	11,759	8,542	3,475	2,107	1,368	8,335	3,109	5,226	30.41	34.02	26.72	10.00	4.00	1.00
Sudan	76,772	38,548	38,223	4,597	2,096	2,502	17,892	7,646	10,246	22.80	24.34	21.36	28.00	6.00	2.00
Syria	39,037	19,188	19,849	2,247	953	1,294	13,742	6,803	6,939	22.11	26.07	18.31	25.00	9.00	1.00
Tunisia	33,906	17,334	16,572	2,550	1,186	1,364	10,246	6,150	4,095	15.73	19.16	12.38	44.00	12.00	5.00
UAE	7,579	6,121	1,459	1,707	1,312	396	2,103	1,089	1,014	18.50	19.8	15.49	40.00	12.00	5.00
Yemen	52,644	27,059	25,585	2,627	1,114	1,512	9,210	4,300	4,910	27.60	30.64	24.76	33.00	6.00	2.00

Diabetes, smoking, high blood pressure, high BMI, stress, high cholesterol levels, poor nutrition, and insufficient physical exercise are all considered risk factors responsible for the incidence of CVDs (24). According to Franklin and Wong, hypertension is the main cause of cardiovascular disease, which worsens with age and may be the world's leading cause of mortality (25).

A cross-sectional study conducted among the local population of 53 cities in Punjab, Pakistan, reported that CVDs impacted 17.5% of the population, with females having a higher incidence rate than males and start occurring at a younger age. An inactive lifestyle, low level of activity and family history of disease could be disease risk factors (26). CVDs are also responsible for 40% and 37% of deaths in Egypt and Saudi Arabia, respectively. A comparative cross-sectional study involved students from two medical of both sexes from Saudi Arabia and Egypt revealed a relatively high prevalence of a sedentary life style, obesity, and abdominal obesity. Saudi students revealed a significantly higher prevalence of obesity while male Egyptian students recorded a significantly higher prevalence of hypertension. Both populations were at an elevated risk of acquiring fatal cardiovascular disease within 10 years (23.9% of Saudi students and 16.7% of Egyptian students) (27). In Iran despite the slight recession in the number of smokers, total cholesterol, and blood pressure, adverse trends in physical activity, unhealthy diet, obesity, and fasting plasma glucose must be addressed immediately at a public health level in order to battle the advancement of CVDs (28).

According to the Global Burden of Disease Study 2019, ischemic heart disease is the most common reason for death in EMR and it is the first reason for death in 19 countries in EMR (Afghanistan, Bahrain, Egypt, Iran, Iraq, Jordan, Kuwait, Lebanon, Libya, Morocco, Oman, Palestine, Qatar, Saudi Arabia, Sudan, Syrian Arab Republic, Tunisia, United Arab Emirates, and Yemen) (13). Globally, on concomitant ischemic heart disease was the leading cause of death in people aged between 30 and 70 years in 146 (83%) countries for men and 98 (55.7%) for women. For men, the risk reached as high as 20% and for women as high as 13% in some countries. Other regions that suffer from this high risk of dying from ischemic heart disease were eastern Europe, central Asia, and south Asia (29). The highest increase in the ischemic heart disease percentage between 2009 and 2019, in the EMR, was reported in UAE (130.6%) followed by Jordan (86.3%) then Djibouti (67.9 %) and Egypt (62.9 %). Stroke is the second reason for death in nine countries (Iraq, Jordan, Kuwait, Lebanon, Libya, Morocco, Palestine, Syrian Arab Republic, and Tunisia). The highest increase in stroke percentage between 2009 and 2019, was reported in UAE (105.2%) followed by Jordan (78.5%) then Djibouti (52.7%) (13).

#### Diabetes Mellitus

**Figure 2:**
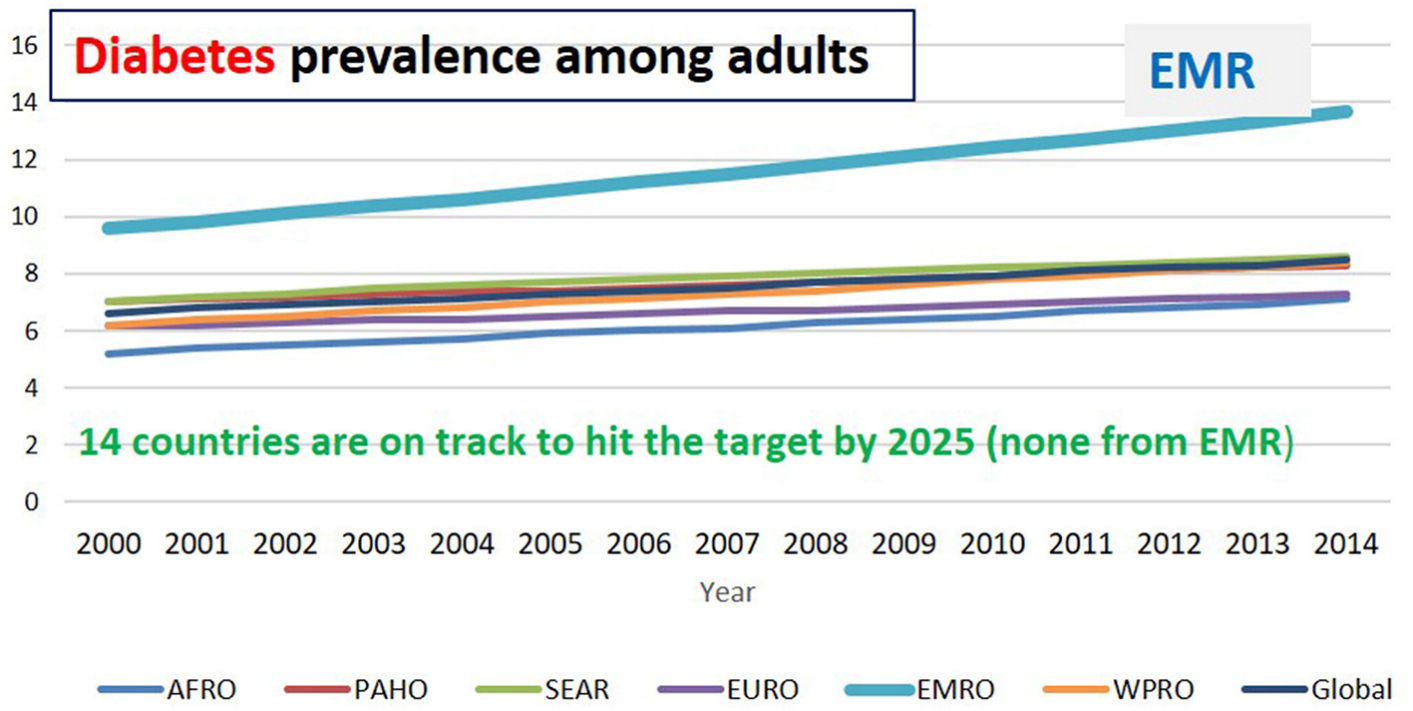
Diabetes prevalence among adults in different regions (6).

In 2019, diabetes mellitus caused 70.9 million (2.8%) of total global DALYs (30). 9.3 percent (463 million people) was the conservative estimate for the prevalence of diabetes in 2019 which is expected to rise by 2030 to 10.2% (578 million) and by 2045 to 10.9% (700 million). The prevalence is higher in urban (10.8%) than rural (7.2%) areas, and in high-income (10.4%) than low-income countries (4.0%). Nearly, half of people (51%) living with diabetes are not aware of that they are diabetics. Impaired glucose tolerance affected 7.5% of the world's population (374 million) in 2019, rising to 8.0% (454 million) by 2030 and 8.6% (548 million) by 2045 (31). Notably, in 2019 the prevalence of diabetes was the highest in the EMR (11.96%) compared with all other regions. Sudan, Qatar, Iran, Bahrain, Somalia, and Djibouti revealed the highest percentage of Diabetes among individuals aged 20–80 years (22.1, 19.9, 17.2, 16.3, 15.8, and 15.6%, respectively) (32) (Table 2; Figure 2).

**Table 2:** Obesity, raised fasting blood glucose, raised blood pressure, diabetes prevalence, and cancer trends in EMR (6).

	**Diabetes (%) Age 20–79**	**Cancer cases (*N*)**	**Cancer Rates/100,000 (Age St.)**	**Cumulative cancer risk**	**Obesity prevalence among adults, BMI** **≥30 (Age St.) (%)**			**Hyper-tension (%) (Age St.)**	**Raised blood pressure (SBP ≥140 Or DBP ≥90)**	**Raised fasting blood glucose (≥7.0) mmol/L**	**Mean total cholesterol (Age St.)**			**Mean non-HDL cholesterol (Age St.)**
	**Both**	**Both**	**Both**	**Both**	**Both**	**M**	**F**	**Both**	**Both**	**Both**	**Both**	**M**	**F**	**Both**
	**2019**	**2020**	**2020**	**2020**	**2016**	**2016**	**2016**	**2019**	**2015**	**2014**	**2018**	**2018**	**2018**	**2018**
Afghanistan	9.2	20,975	175.4	20	5.5	3.2	7.6	33.7	30.6	11.9	4.1	4.0	4.2	3
Bahrain	16.3	1,177	180.6	26.66	29.8	25.5	36.8	28.6	21.4	11.5	4.5	4.5	4.6	3.3
Djibouti	15.6	737	146.6	15.21	13.5	8.6	18.3	24.1	26.8	8.1	4.2	4.1	4.3	2.8
Egypt	5.1	129,577	258	31.39	32	22.7	41.1	33.3	25	17.9	4.4	4.2	4.5	3.3
Iran	17.2	127,548	245.2	35.6	25.8	19.3	32.2	23.6	19.7	12.1	4.4	4.3	4.5	3.1
Iraq	9.6	31,801	217.6	25.86	30.4	23.4	37	40.7	25.2	17.4	4.7	4.6	4.7	3.4
Jordan	8.8	11,107	251.8	29.63	35.5	28.2	43.1	33.2	21	16.8	4.8	4.7	4.9	3.5
Kuwait	12.7	3,716	185.3	27.97	37.9	33.3	45.6	33.5	23.6	19.6	4.8	4.8	4.9	3.5
Lebanon	12.2	11,287	252.5	30.53	32	27.4	37	32.2	20.7	13.4	5.0	5.0	5.0	3.7
Libya	11.2	7,388	212.8	28.17	32.5	25	39.6	34.2	23.7	15.9	4.3	4.2	4.4	3
Morocco	10.2	57,772	238.8	26.96	26.1	19.4	32.2	29.5	26.1	13.7	4.1	4.0	4.2	2.8
Oman	7	3,557	165.4	17.58	27	22.9	33.7	38.6	24.8	13.5	4.6	4.6	4.7	3.4
Pakistan	10.1	170,668	178.7	19.81	8.6	6	11.3	34.2	30.5	12.4	4.2	4.1	4.3	3.1
Qatar	19.9	1,435	172.2	28.55	35.1	32.5	43.1	31.1	22.4	18.9	4.3	4.3	4.5	2.9
Saudi Arabia	15.6	26,505	152.1	20.13	35.4	30.8	42.3	31.9	23.3	17.4	4.6	4.5	4.6	3.3
Somalia	15.8	9,140	189.7	20.24	8.3	3.9	12.3	27.1	32.9	6.8	4.2	4.1	4.3	3.1
Sudan	22.1	25,347	153.4	17.85	8.6	3.8	12.4	27.4	30.2	8.9	4.1	3.9	4.3	3
Syria	5.1	20,193	241.5	28.61	27.8	20.9	34.8	33.5	24.5	14.6	4.5	4.5	4.6	3.3
Tunisia	13.5	19,031	214.9	28.05	26.9	19.1	34.3	32.1	23.2	12.5	4.3	4.1	4.5	3
UAE	8.5	4,611	170.7	28.49	31.7	27.5	41	27.7	21.1	15.1	4.6	4.5	4.7	3.4
Yemen	5.4	14,848	154.4	21.81	17.1	12	22	34.8	30.7	11.3	4.5	4.4	4.6	3.4

In EMR, the total number of fatalities due to diabetes was 186,841 thousand in 2019. Pakistan recorded the highest number (80,976) followed by Egypt (26,844), Iran (157,018) then Morocco (17,947) (6) (Table 1). According to the 2019 Global Burden of Disease Study, diabetes is the second cause of death in two countries (Bahrain and Jordan) and the third reason for death in three countries (Iraq, Palestine, and Qatar) in the region. The highest increase in diabetes percentage between 2009 and 2019 in EMR was reported in UAE (124.2%) followed by Palestine (89.1%) then Bahrain (88.1%), Iran (80.8%), and Jourdan (69.7%) (13).

The mortality rate due to diabetes in Bahrain was 14% in 2016. A cross sectional study reported that type 2 diabetes exerts a significant pressure on Bahrain's healthcare system—primarily due to costly diabetes-related complications. Thereby, reducing the risk factors for diabetes is mandatory to minimize disabling and expensive complications (33). Additionally, multivariate analysis for a wide community-based survey in Pakistan using glycated hemoglobin revealed a significant link between type 2 diabetes and old age. Increase in body mass index, central obesity, positive family history, and having hypertension with type 2 diabetes were inversely related to education (34).

Egypt has been identified by the International Diabetes Federation as the ninth leading country in the world for the number of type 2 diabetes patients. The frequency of type 2 diabetes has nearly tripled in the last two decades in Egypt. This dramatic increase could be due to an increase in the typical risk factors for type 2 diabetes, such as obesity and physical inactivity, as well as a shift in dietary habits, or to other risk factors specific to Egypt. Increased exposure to environmental risk factors such as pesticides and a higher prevalence of chronic hepatitis C are two examples (35).

In a population-level mathematical model among Qatari, the baseline scenario revealed that type 2 diabetes prevalence would be upregulated from 16.7% in 2016 to 24.0% in 2050. By lowering obesity prevalence by 10–50%, type 2 diabetes prevalence would reduced by 7.8–33.7%, while by reducing physical inactivity prevalence by 10–50%, type 2 diabetes prevalence would reduced by 0.5–6.9% by 2050 (36).

#### Cancer

Globally in 2019, total cancers recorded 23.6 million incident cases, 10.0 million deaths, and 250 million DALYs. Total cancers were the second-ruling reason for death and DALYs in 2019 worldwide (13). Globally in 2020, an estimated 19.3 million new cancer cases (18.1 million excluding non-melanoma skin cancer) and almost 10.0 million cancer deaths (9.9 million excluding non-melanoma skin cancer) occurred (37). According to long-term projections, the EMR countries will suffer from a disturbing rise in the number of cancer patients reaching a 1.8 fold by 2030 (38). The highest number of cancer cases in EMR in 2020 has been recorded in Pakistan (170,668) thousand individuals followed by Egypt (129,577), then Iran (127,548) (9) (Table 2). Bahrain, Qatar, Iran, and Lebanon reported a 16% mortality rate due to cancers, Kuwait reported 15% while Egypt reported 13% (4).

Bahrain, which is among the high income gulf countries, suffers from a rising burden of cancer (39, 40). Breast, colorectal, and lung cancers, followed by non-Hodgkin lymphoma and leukemia, are the five most frequently diagnosed cancers in Bahrain (41). Obesity, smoking, a sedentary lifestyle, and a high-fat/low-fiber diet are among the significant risk factors for colorectal cancer in Bahrain. Nearly one-third of the population of Bahrain is overweight or obese (42, 43).

A systematic review investigating the epidemiological aspects of gastric cancer in Iran based on articles published during the years 1970–2020 showed that poor levels of economic position and food insecurity raised the probabilities of stomach cancer by 2.42- and 2.57-times, respectively. Moreover, there was a link between dairy products, processed red meat, fruit juice, legumes, smoked and salty fish, salt, strong as well as hot tea consumption with the risk of stomach cancer. There was also an inverse link between fresh fruit, citrus, and garlic consumption and stomach cancer (44).

The global age-standardized rate as reported by the American Institute for Cancer Research, 2018 for all cancers (including non-melanoma skin cancer) for both genders was 197.9 per 100,000 in 2018. Men revealed a higher rate (218.6 per 100,000) than women (182.6 per 100,000) (45). Most of the EMR countries revealed a relatively high rate of cancer incidence as nine countries in the region have cancer rates of more than (200 per 100,000). The highest cancer rates as revealed in 2020 have been reported in Egypt (258 per 100,000) followed by Lebanon (252.5 per 100,000) then Jordan (251.8 per 100,000) then Iran (245.2 per100,000) followed by Syria (241.5 per 100,000), and then Morocco (238.8 per 100,000) (9) (Table 2).

In EMR, the total number of fatalities due to cancer (431,312) in 2019. Pakistan recorded the highest number (124,328) followed by Egypt (85,226), Iran (61,063), then Morocco (33,845) (6) (Table 1). By 2050, a three-fold increase in cancer incidence relative to 2013 was estimated to occur in Egypt (46). The highest increase in cancer percentage between 2009 and 2019 in EMR was reported in the UAE (241.7% increase in pancreatic cancer) followed by Jordan and Qatar (103.7 and 95.2%, respectively increase in lung cancer) (13).

### Risk Factors Burden

A dramatic increase in NCDs-related risk factors has been reported in the EMR in the past 10 years (13). The risk factors of NCDs comprise metabolic-physiological-related conditions (including obesity, high blood pressure, high fasting plasma glucose, high blood cholesterol) as well as behavioral-related activities (including smoking, low physical activity, unhealthy diet consumption, excessive use of alcohol). An analytic review published in 2019 reported that individuals who followed healthy lifestyle practices including regular physical activity, sound nutrition, weight management, and non-smoking revealed a significant downregulation of CVDs risk by >80% and diabetes by >90% (47). Additionally, another study outlined that around 40% of cancer cases could be prevented by reducing exposure to cancer risk factors including diet, nutrition, and physical activity (45).

### Dietary Risks or Unhealthy Diet Consumption

Dietary risk is defined as eating a diet low in whole grains, nuts, seeds, fruit, vegetables, fibers, legumes, omega-3 fatty acids, PUFA, milk, and calcium as well as a diet high in sodium, trans fats, red or processed meat, and sugar-sweetened beverages (SSB). Globally in 2019, dietary risks were responsible for 188 million DALYs and 7.94 million deaths among adults aged 25 and older. It was the fifth-ruling risk factor for attributable DALYs (48). Dietary risk is the third risk factor in Syria and the fourth risk factor in 6 countries in the EMR (Afghanistan, Morocco, Oman, Pakistan, KSA, and Yemen) responsible for the most deaths and disabilities. The highest increase in dietary risk percentage between 2009 and 2019 in EMR was reported in UAE (136.9%), followed by Jordan (84.7%) then Qatar (66.8%) (13), as shown in **Table 5**.

#### Fruits and Vegetables

An adequate daily intake of fruits and vegetables is associated with reduced risks of CVDs (49), stroke (50), type 2 diabetes (51), and certain types of cancer (52, 53), which are the major causes of mortality and morbidity in the EMR. The 2002 Joint FAO/WHO Expert Consultation on Diet, Nutrition and the Prevention of Chronic Diseases recommends a minimum of 400 g per day of fruits and vegetables, an equivalent of ≥5 servings of fruits and vegetables per day, excluding starchy roots (54).

In 2013, the rate of fruits and vegetables intake among individuals living in the EMR was 280 g per day, which is lower than WHO recommendation for the prevention of NCDs. Furthermore, it has been reported that the mean daily intake of fruits in the Middle East and North Africa region was <130 g per day, and the mean intake of vegetables was less than 200 g per day (2). According to the food balance sheets 2019, the mean fruits and vegetables intake among EMR countries is 32 kg/capita/year (10), see Table 3. It is noteworthy, that data concerning fruits and vegetables intake in EMR are limited.

**Table 3:** Food consumption in EMR (2019) (10).

	**Legume **	**Eggs**	**Fruits and**		**Fish**		**Meat**	**Milk**	**Dietary oils and fats**										**Carbohydrates**				**Honey, sugar and**		
			**vegetables**																				**sweeteners**		
	**Beans**	**Eggs**	**Vegetables**	**Fruits**	**Fresh water fish**	**Marine fish**			**Fats, animals**	**Nuts**	**Butter**	**Coconut oil**	**Olive oil **	**Cotton seed oil**	**Palm oil**	**Sesame oil **	**Soybean oil**	**Sunflower oil**	**Potatoes**	**Rice**	**Sweet potatoes**	**Wheat**	**Honey**	**Sugar raw **	**sweeteners**
Afghanistan		0.82	54.58	6.64	0.24		0.31	38.04	0.23	1.44	1.06	0.01	0.02	0.1	4.46	0.04	0.29	1.47	25.7	15.16	0	161.9	0.04	17.08	0.54
Bahrain																									
Djibouti	1.97	0.98	47.51	12.83	0.02	0.27	0.72	19.58	0.48	0.04	0.15	0.18	0.15		9.6	0	0.83	0.58	16.65	49.38	0	121.8	0.02	41.18	3.46
Egypt	0.5	2.85	66.16	24.01	13.55	0.14	0.8	24.8	0.22	0.39	1.31	0.05	0.12	0.01	0		3.23	2	28.38	50.92	2.97	145.9	0.03	25.89	0.73
Iran	1.57	8.33	69.35	54.69	5.86	0.39	0.09	23.24	0.18	9.81	2.7	0.09	0.14	0.02		0.17	6.38	4.68	31.1	40.29		156	0.86	27.22	3.64
Iraq	1.63	10.16	54.75	22.24	2.4	0.13	0.03	26.22	0.18	1.32	0.24	0.02	0.05	0.01	4.25	0.12	1.13	6.97	18.08	50.89	0	135.8	0.02	17.3	3.69
Jordan	0.32	5.3	57.27	5	0.56	0.9	0.06	48.19	1.26	1.78	0.28	0.06	2.18		3.86	0.81	3.77	6.78	20.77	20.27	0.12	96.7	0.11	29.94	8.71
Kuwait	0.3	14.2	100.3	13.31	2.55	1.56	2.17	39.38	0.04	2.34	2.08		1.05	0.02	4.24	0.03	2.46	0.61	38.71	51.98	0.2	102.1	0.24	34.52	1.61
Lebanon	1.03	5.83	61.23	30.73	1.33	1.48	0	76.92	0.49	5.52	0.76	0	2.21	0.01		0.87	6.9	4.93	41.4	12.49	0.31	123.9	0.23	39.68	3.54
Libya	0.61	9.29	79.23	29.91	0.04	0.21	0.87	49.19	0.2	5.75	0.3	0.03	1.9	0.27	0	0	1.3	3.21	25.21	22.47	0	129.4	0.23	28.13	16.3
Morocco	0.12	8.69	58.54	23.48	0.49	1.05	1.9	25.42	0.55	3.23	1.42	0.01	4.02			0	6.04	1.52	41.34	2.1	0.21	176.9	0.18	33.51	0.84
Oman	0.25	8.99	103.53	45.59	0.47	3.04	2.8	86.64	0.76	1.23	0.9	0.18	0.04		6.03	0.03	0.56	1.08	21.27	51.13	0.03	74.9	0.38	20.66	10.21
Pakistan	0.97	3.47	13.7	23.02	1.23	0	0	113.6	0.93	0.31	5.21	0	0.01	0.27	4.08	0	1.2	0	14.74	19.73	0.07	103.9	0.02	21.06	0.3
Qatar																									
KSA	0.24	8.72	42.04	7.94	4.26	0.37	1.55	44.84	0.52	1.37	1.19	0.1	0.6	0.43	11.36	0.7	0.57	1.73	16.99	53.25	0.15	97.6	0.38	30.27	1.67
Somalia																									
Sudan	0.18	1.36	27.51	20.64	0.97	0.05	3.41	92.57	0.61		0.01	0	0.01	0.29	0.58	0.54	0	2.18	9.31	1.23	5.57	61.6	0.02	28.21	0.44
Syria	0.29	9.43	76.79	20.65	0.41	0.04	0.13	82.76	0.99	6.73	0.5	0.06	5.94	0.1	0.31	0.17	1.68	7.2	26.91	10.46	0	132.9	0.13	20.91	13.75
Tunisia	0.43	7.15	159.76	30.3	0.28	0.43	0.28	91.32	0.64	6.47	0.82	0.28	3.92		2.77	0.02	3.85	1.35	29.09	1.53	0	198.5	0.29	33.06	0.76
UAE																									
Yemen	0.69	1.5	10.17	13.76			0.09	10.13	0.33	0.39	0.04	0.01	0.04	0.05	5.21	0.23	0.02	0.36	6.15	28.61	0.01	115.2	0.06	24.35	1.5

Most individuals living in the EMR have an insufficient intake of fruits and vegetables. It has been established that only 7.3% of individuals from Saudi Arabia aged 15–64 years were consuming the WHO-recommended five servings of fruits and vegetables per day and only 2.6% met the CDC guidelines for daily consumption of fruits and vegetables (55). In a more recent cross-sectional study conducted on 1,437 individuals, aged ≥ 18 years, 88% of the subjects recorded low intake of fruits and vegetables with a significant increase in fast food consumption (56). The relationship between food consumption patterns and expenditure was investigated in village Kabal in rural areas of Pakistan, using a sample size of 100 households. The study outlined that an adult consumes nearly 74.68 g of meat, 166.34 g of milk, 372.51 g of flour, 70.29 g of rice, 28.31 g of pulses, 177.12 g of vegetables, 66.39 g of fruits, 6.76 g of black tea, 53.60 g of fats, and 73.21 g of sugar daily (57). Furthermore, an assessment of fruits and vegetables consumption among 473 medical students in Egypt outlined that 8.2% of students knew the recommended five daily servings for fruits and vegetables, and 23.26% consumed the five daily servings. Healthy food items were tried by only 35.7% of students (58).

#### Fat Consumption

Fat consists of trans-fatty acids (TFAs), saturated fatty acids (SFA), and unsaturated fatty acids (59). Saturated fatty acids can be found in animal products like milk, butter, cheese, as well as most plant oils, particularly palm and coconut oil, which are high in SFA. Lauric acid, myristic acid, and palmitic acid (PA) are all major sources of SFA, and they all raise low-density lipoprotein cholesterol (LDL-c) (60). Increased inflammation, oxidative stress, and decreased nitric oxide and insulin signaling is some of the impacts of PA, which is found in palm oil (61, 62). The American Heart Association recommends a healthy dietary pattern that achieves 5–6% of calories from saturated fat (about 13 g of saturated fat per day). In EMR, three countries have exceeded 13% of energy from saturated fats, Djibouti (15.2% of energy) followed by Yemen (13.9% of energy), and KSA (13.5% of energy) (63) (see Table 4). In Saudi Arabia, a significant positive association was found between the intake of fats, protein, and calories and the risk of breast cancer. Adjusted odds ratios for the highest quartile of intake versus the lowest were 1.88 for cholesterol, 2.12 for polyunsaturated fat, 2.25 for animal protein, 2.43 for saturated fat, and 2.69 for total energy from dietary intake (64).

**Table 4:** Salt, fat, and sugar-sweetened beverage consumption in EMR (4, 11, 12, 63).

	**Salt**						**Dietary fats and oils**						**Sugar-Sweetened Beverages**								
	**Estimated sodium**			**Mean population salt**			**Saturated**	**Omega-6**	**Trans fat**	**Dietary**	**Seafood**	**Plant**	**Mean juice intake**			**Mean SSB intake**			**Mean milk intake**		
	**intakes (g/day) in 2010**			**intake, adults (g/day) in 2016**			**fat**	**PUFA**	**fat**	**cholesterol**	**omega-3**	**omega-3**	**(servings/day)**			**(servings/day)**			**(servings/day)**		
							**(% energy)**	**(% energy)**	**(% energy)**	**(mg/d)**	**fat (mg/d)**	**fat (mg/d)**									
	**Both**	**M**	**F**	**Total**	**Total**	**Total**	**Total**	**Total**	**Total**	**Total**	**Total**	**Total**	**F**	**M**	**Both**	**F**	**M**	**Both**	**F**	**M**	**Both**
Afghanistan	3.39	3.55	3.22	9	8	9	10.8	4.6	1.3	212	30	665	0.03	0.02	0.03	0.34	0.37	0.36	0.36	0.34	0.35
Bahrain	5.38	5.57	5.05	14	13	14	8.9	6.4	3.2	245	52	1,451	0.26	0.22	0.24	0.50	0.53	0.51	0.79	0.70	0.75
Djibouti	2.36	2.48	2.24	6	6	6	15.2	3.7	0.7	213	13	640	0.02	0.02	0.02	0.75	0.81	0.78	0.49	0.45	0.47
Egypt	3.68	3.85	3.52	10	9	9	9.6	4.8	6.5	402	42	568	0.14	0.12	0.13	0.32	0.34	0.33	0.61	0.55	0.58
Iran	4.02	4.21	3.83	11	10	10	10.8	6.2	2.4	232	77	1,195	0.34	0.28	0.31	0.17	0.17	0.17	0.62	0.56	0.59
Iraq	3.76	3.95	3.59	10	9	10	10.3	4.4	1.7	288	50	922	0.14	0.11	0.13	0.37	0.40	0.38	0.52	0.44	0.48
Jordan	4.13	4.31	3.95	11	10	10	11.3	6.6	1.6	239	44	1,410	0.15	0.13	0.14	0.60	0.67	0.64	0.72	0.65	0.68
Kuwait	3.88	4.01	3.65	10	9	10	12.8	5.7	1.8	214	47	1,159	0.11	0.09	0.10	0.42	0.46	0.44	0.69	0.64	0.66
Lebanon	3.13	3.3	2.98	8	8	8	11.2	9.9	1.6	287	76	1,918	0.20	0.17	0.18	0.69	0.74	0.72	0.33	0.29	0.31
Libya	4.24	4.45	4.03	11	10	11	7.4	7.8	1.6	194	8	1,265	0.16	0.14	0.15	0.40	0.44	0.42	0.63	0.57	0.60
Morocco	4.31	4.53	4.11	12	10	11	9	6.4	1	239	60	1,283	0.13	0.10	0.12	0.46	0.50	0.48	0.29	0.26	0.28
Oman	3.78	3.93	3.56	10	9	10	10.3	5.9	1.8	253	45	1,181	0.16	0.14	0.15	0.40	0.43	0.42	0.56	0.50	0.53
Pakistan	3.91	4.05	3.75	10	10	10	3.8	3.5	5.8	157	16	277	0.05	0.04	0.04	0.45	0.50	0.48	0.54	0.50	0.52
Palestine	3.86	4.04	3.69	_	_	_	8.6	5.2	1.9	258	63	973	0.16	0.14	0.15	0.43	0.46	0.44	0.62	0.56	0.59
Qatar	4.21	4.29	3.9	11	10	11	10.2	6.8	1.7	220	8	1,505	0.23	0.19	0.21	0.48	0.51	0.50	0.73	0.65	0.69
KSA	3.2	3.33	3.03	8	8	8	13.5	4.1	1.1	286	50	656	0.37	0.31	0.34	0.38	0.42	0.40	0.68	0.61	0.64
Somalia	2.07	2.17	1.97	6	5	5	10.9	3.8	0.7	170	48	337	0.01	0.01	0.01	0.23	0.26	0.25	0.17	0.15	0.16
Sudan	2.37	2.49	2.26	_	_	8.2*	9.1	4.7	1.1	278	1,291	526	0.05	0.04	0.05	0.59	0.66	0.62	1.03	0.93	0.98
Syria	4.18	4.37	3.99	11	10	11	9.7	5.6	1.5	251	58	1,134	0.20	0.16	0.18	0.50	0.54	0.52	0.81	0.73	0.77
Tunisia	4.43	4.63	4.24	12	11	11	9.8	7.5	1	286	17	2,215	0.12	0.10	0.11	0.40	0.43	0.42	0.63	0.58	0.61
UAE	3.67	3.76	3.43	10	9	9	10.7	5.2	1.1	262	377	962	0.28	0.23	0.25	0.43	0.47	0.45	0.73	0.67	0.70
Yemen	3.37	3.55	3.21	9	8	9	13.9	3.6	1.5	224	52	362	0.12	0.09	0.10	0.44	0.49	0.46	0.34	0.30	0.32

A diet high in trans-fatty acids is defined as any intake (in percentage daily energy) of trans fat from all sources, primarily partially hydrogenated vegetable oils and ruminant products. TFAs are typically found in processed food, fast food, snack food, fried food, pies, cookies, margarine, and spreads (59). In 2019, a diet high in TFAs was responsible for 14.2 million DALYs and 645,000 deaths. It was the seventh-ruling dietary risk factor for attributable DALYs (65).

The consumption of TFAs increases the risk of death from any cause by 34% and coronary heart disease by 28% (66). An increase in coronary heart disease mortality estimated by 12% occurs as a result of every 1% increase in daily energy obtained from TFAs (67). Industrial TFAs intake has also been related to an increased risk for other NCDs and associated conditions such as ovarian cancer (68), infertility, endometriosis, Alzheimer's disease, diabetes, and obesity (59, 69). Higher consumption of hydrogenated vegetable oils was associated with an increased risk of myocardial infarction in a cohort study conducted among an Iranian population (70).

Despite WHO recommendations that total trans fat intake should not exceed 1% of total energy intake, which translates to >2.2 g/day for a 2,000-calorie diet (71), in 2010, four countries in the region have exceeded this level (Iran, Bahrain, Pakistan, and Egypt) (63) (Table 4). Laboratory analysis was conducted for profiling TFAs, saturated, and unsaturated fatty acids in the products that are mostly consumed in the major governorates in Egypt. On average, 34% of the products exceeded the TFAs limit (more than 2 g TFA/100 g of fat). The study revealed that around one third of products in the Egyptian market have a high TFAs content (72). Iran has achieved a marked improvement in the reduction of TFAs as early studies recorded 12.3 g as a mean intake in 2007, while in 2013 this has been reduced to 1.42 and 1.5 g in 2018 (73–76).

The 2019 American College of Cardiology/American Heart Association Guideline on the Primary Prevention of Cardiovascular Disease concluded that a diet containing reduced amounts of cholesterol and sodium could be beneficial to decrease atherosclerotic CVDs risk (77). Every increase in dietary cholesterol by 100 mg/day predicted an increase in LDL-c from 1.90 to 4.58 mg/dl depending on the model (78). The 2015 National Lipid Association Recommendations for Patient-Centered Management of Dyslipidemia, recommend limiting dietary cholesterol to <200 mg/d to lower LDL-c and non–high-density lipoprotein cholesterol (HDL-c) concentrations, however, insufficient evidence among populations doesn't exist (79). Within EMR, all of the countries are beyond the previous recommended level, particularly Egypt where the recorded dietary cholesterol level was 402 mg/day, followed by Iraq 288 mg/day, then Lebanon 287 mg/day, then KSA and Tunisia 286 mg/day (63) (Table 4).

#### High Sugar Diet

The term “sugars” includes intrinsic sugars, from intact fruit and vegetables; milk, as well as free sugars, which are added to foods and beverages, and sugars naturally present in honey, syrups, fruit juices, and fruit juice concentrates (80).

There is uprising worry regarding the free sugars' intake, particularly in the form of SSB that increases the overall energy consumption and may reduce healthy food items' intake. This leads to unhealthy dietary habits, subsequent weight gain, and increased risk of NCDs (54, 81–83). Another concern is the association between intake of free sugars and dental caries (54, 84–86). Dental diseases are the most prevalent NCDs globally (87, 88).

The established dietary goal for free sugars' intake is <10% of total energy but ideally less than 5% of total energy intake. This 10% ratio is equivalent to 50 g for a person of healthy body weight consuming about 2,000 calories per day (54).

#### Juice and SSB Consumption

A diet high in SSB is defined as any intake (in grams per day) of beverages with ≥50 kcal per 226.8 g serving, including sodas, carbonated beverages, energy drinks, and fruit drinks, but excluding 100% fruit and vegetable juices. In 2019, a diet high in sugar-sweetened beverages was responsible for 6.31 million DALYs and 242 000 deaths. It was the 13th-leading dietary risk factor for DALYs (89).

The average consumption of raw sugar in EMR is 80 g per day, while the recommended amount of sugar is equivalent to 50 g. The highest mean consumption of SSB among EMR countries has been recorded in Djibouti 0.78 serving/day followed by Lebanon 0.72, then Jordan 0.64, then Sudan 0.62, then Syria 0.52, and Bahrain 0.51. The highest juice intake in EMR has been recorded in KSA 0.34 serving/day followed by Iran 0.31, then UAE 0.25 and Bahrain 0.24 (12) (as outlined in Table 4).

A review of the literature reveals that SSBs contribute partly to the obesity epidemic, as reported by epidemiologic studies, which emphasized the link between SSB consumption and long-term weight gain, type 2 diabetes mellitus, and CVDs risk. It is hypothesized that SSB contribute to weight gain due to their high added sugar content, low satiety, and potential partial compensation for total energy leading to increased energy intake (90, 91). In addition, because of their large consumed quantities besides their high contents of rapidly absorbable carbohydrates such as different forms of sugar and high-fructose corn syrup, SSB could be responsible for increased type 2 diabetes mellitus and CVDs incidence. Independent of obesity, SSB could serve as a contributor to a high dietary glycemic load leading to inflammation, insulin resistance, and impaired ß-cell function (92). Fructose from any sugar or high-fructose corn syrup may also increase blood pressure, and enhance the cumulative effects of visceral adiposity, dyslipidemia, and ectopic fat precipitation due to upregulated hepatic *de novo* lipogenesis (93).

#### Salt/Sodium Intake

Salt consumption within the WHO-recommended level for adults is <5 g per person per day (2 g per day of sodium). Excessive salt consumption is linked to adverse health outcomes, such as the increased risk of hypertension (raised blood pressure), which in turn leads to stroke and heart disease (94). The current salt intake in the Region averages more than 10 g per person per day, which is double the recommended level set by WHO. In 2010, within EMR countries the highest mean salt intake has been recorded in Bahrain (14 g/day) followed by Libya, Morocco, Qatar, Syria, and Tunisia (11 g/day) (11) (see Table 4). Conversely, according to more recently collected data based on urinary excretion, the highest level of salt intake was observed in Morocco (10.6 g/day), while the lowest was observed in Lebanon (5.6 g/day) and the UAE (6.8 g/day) (19). Based on dietary assessment questionnaires, the highest levels of salt intake were observed amongst Iranian children and adolescents (14.3–16.2 g/day) and adults in Bahrain (9.3–13.3 g/day) and Lebanon (10.9 g/day). Per capita estimates were also high in Oman (11.5 g/day) and Tunisia (10.2 g/day) (19). Sodium is an essential nutrient necessary for the maintenance of plasma volume, acid-base balance, the transmission of nerve impulses, and normal cell function (95). In our diet, the main source of sodium is salt, despite it can be attained from sodium glutamate, used as a food additive in many processed foods (95). In 2019, a diet high in sodium (more than 3 g) was responsible for 44.9 million DALYs and 1.89 million deaths. It was the leading dietary risk factor for causing DALYs (96). The highest mean sodium intake has been recorded in Bahrain (5.8 g/day) followed by Tunisia (4.43 g/day), then Morocco (4.31 g/day), Libya (4.24 g/day), Qatar (4.21 g/day), Syria (4.18 g/day), and Jordan (4.13 g/day) (11) (see Table 4).

The EMR population should be aware of how much salt they consume as the disease burden of CVDs, resulting mainly due to salt and subsequent high blood pressure, is very high in the region (97). In a recent study, the salt intake levels were estimated in 15 out of the 22 countries in EMR, national salt reduction initiatives were identified in 13 countries including Bahrain, Egypt, Iran, Jordan, KSA, Kuwait, Lebanon, Morocco, Oman, Palestine, Qatar, Tunisia, and the UAE. The majority of countries were discovered to be implementing complex reduction measures, which included two or more implementation strategies. Taxation was the least popular implementation option, whereas reformulation was the most popular (100%), followed by consumer education (77%), initiatives in specialized situations (54%), and front-of-pack labeling (46%) (19).

### Obesity

**Figure 3:**
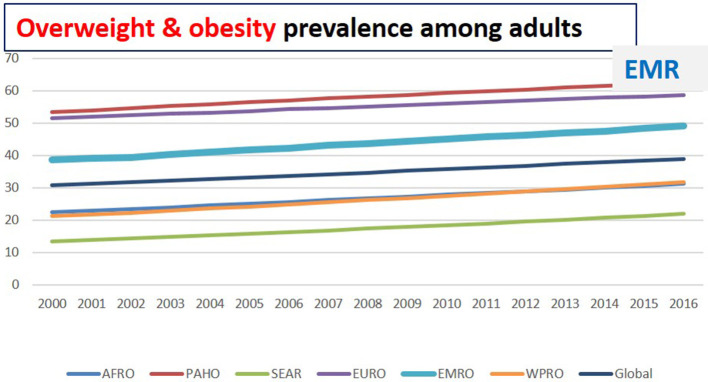
Overweight and obesity prevalence among adults in different regions (6).

The prevalence of obesity (BMI ≥ 30 kg/m^2^) has almost tripled worldwide since 1975. There were 650 million obese adults aged 18 years in 2016, with a global prevalence of nearly 13%. High body-mass index (BMI) was responsible for 160 million DALYs and 5.02 million deaths in 2019. It was the seventh-ruling risk factor for attributable DALYs in 2019 (98). Being obese is usually linked to an increased risk of hypertension and many NCDs (including diabetes, CVDs, and cancers) (99). Shifts in eating behavior toward diets containing energy-dense foods, high in fat and sugars, and less physical activity due to the sedentary nature of many forms of work and modes of transportation are contributing to the rise in obesity. The prevalence of obesity in the EMR is the third-highest across all global regions (4). The current prevalence of obesity is estimated at 25.1%, while the prevalence of overweight is around 56.41%. Among the EMR, the gulf countries revealed the highest rate of obesity. The highest prevalence of obesity in EMR has been reported in Kuwait (37.9%), Jordan (35.5%), Saudi Arabia (35.4%), Qatar (35.1), Libya (32.5%), Egypt (32%), Lebanon (32%), and UAE (31.7%). According to the latest estimates, the prevalence of excess BMI in adults in EMR has increased by 3% between 2012 and 2016 (6) (Table 2; Figure 3).

The high prevalence of people who are overweight or have obesity in Saudi Arabia is considered a public health concern, as revealed in a cross-sectional study carried out on a representative sample of 1,681 adult patients. Being overweight and having obesity were found to be prevalent in 38.3% and 27.6% of the population, respectively. Obesity was not shown to be connected with smoking, although it was found to be associated with hypertension. The risk of overweight or obesity was significantly inversely correlated with the monthly income (100).

The most recent national survey conducted in Egypt revealed that 39.8% of adult Egyptians suffered from obesity with a more prevalent in adult females than males, nearly 25% have normal BMI while the rest are either obese or overweight (101). A study analyzing the health effects of being overweight and having obesity conducted over 25 years in 195 countries, revealed that 19 million Egyptians suffer from obesity, representing 35% of all adults, which is the highest rate in the world. Moreover, the study outlined that 3.6 million children (10.2% of Egyptian children) suffer from obesity (102).

Research published in 2020 indicated that almost three-quarters of men and women in Jordan were overweight or obese. Obesity rates in men were around twice as high in 2017 as they were in 2009. In the multivariate analysis, age, region of residence, and marital status were significantly associated with obesity in both genders. Obesity was significantly linked with increased odds of diabetes mellitus, hypertension, elevated triglycerides, and low high-density lipoprotein cholesterol after adjusting for age (103).

**Figure 4:**
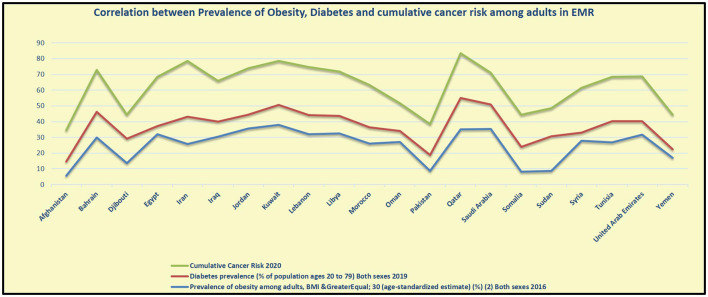
Correlation between the prevalence of obesity, diabetes, and cumulative cancer risk among adults in EMR (6, 26).

**Figure 5:**
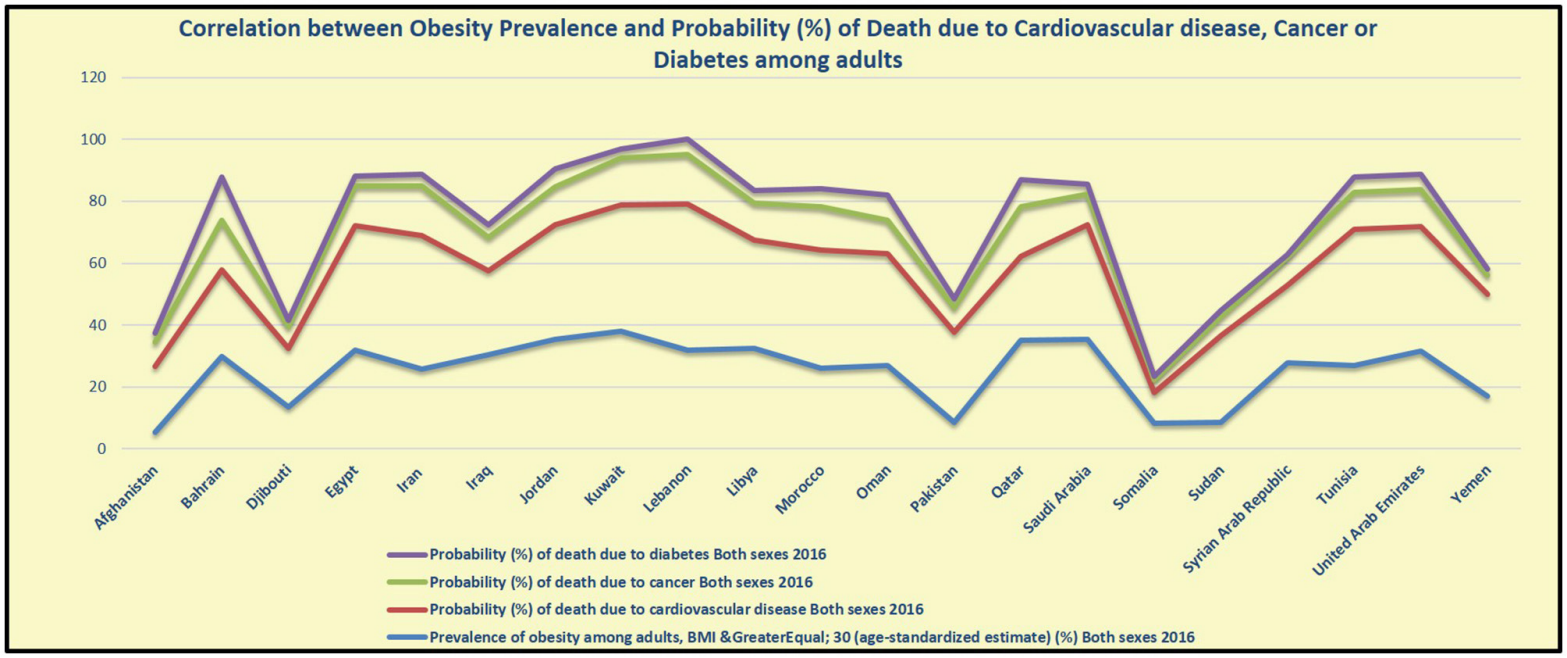
Correlation between the prevalence of obesity, probability of death due to CVDs, cancers, and diabetes among adults in EMR (6).

Ultimately, obesity is the first reported risk factor responsible for the total number of DALYs in 2019 in eight countries in the region (Bahrain, Jordan, Kuwait, Libya, Oman, Qatar, Saudi Arabia, and UAE). It is the second reported risk factor in the other seven countries (Egypt, Iran, Iraq, Morocco, Palestine, Syria, and Tunisia). The highest increase in obesity percentage between 2009 and 2019 in EMR was reported in UAE (133.4%) followed by Djibouti (106.5), then Jordan (96.3%), Qatar (88%), Bahrain (86.9%), and Afghanistan (80.1%). The dramatic increase in obesity involves low-income countries in the region also including Djibouti and Afghanistan (13) (as indicated in Table 5). The prevalence figures revealed that obesity constitutes a significant public health concern in EMR because of its significant correlation to NCDs (see Figures 4, 5).

**Table 5:** The rank of the nutrition related risk factors that causes deaths in 2019 and the percentage change between 2009–2019 (13).

	**High body mass index**	**High blood pressure**	**Malnutrition**	**High fasting plasma glucose**	**Dietary risks**	**High LDL**	**Low physical activity**
Afghanistan	80.1	25.1	−23	39	23.4	31.1	
Bahrain	86.9	55.7	−16.2	93.8	61.8	48.6	96.1
Djibouti	106.5	56.9	−31.5	63.2	52.7		
Egypt	41.6	26.9	−46.8	72.1	31.7	28.7	
Iran	45.3	24.2	−57.4	56	26.1	20	
Iraq	40.5	35	−44.9	47.4	28.7	28	50.9
Jordan	96.3	87.8	11.2	85.9	84.7	86.9	
Kuwait	66.3	37	7.5	63.4	47	42	
Lebanon	38	25.1	−25.9	30.6	22.8	26.3	34
Libya	54.4	49.9	−34.5	64.8	61.5	57	
Morocco	46.1	25.5	−53.2	53.9	29.4	27.6	
Oman	41.1	14.3	21.4	43.1	18.3	14.5	
Pakistan	53	38.4	17	41	28.6	31.1	
Palestine	64.6	32.6	−47.6	57.3	40.1	39.3	
Qatar	88	62.3	21.7	85.9	66.8	51.9	
Saudi Arabia	56.3	29.4	−41.8	53.3	37	39.1	71.4
Somalia		36.1	−7.1	39.7	32.6		
Sudan	51.1	21.1	−41.2	41.6	19.6	26.1	
Syria	23.5	24.9	−57.5	44.8	23.7	20.2	
Tunisia	41.2	25.8	−45.5	43.2	24.7	24.5	
UAE	133.4	140.1		147	136.9	141.5	
Yemen	64.2	43.4	−29.2	66.8	47.4	47.4	
**Color code**							
**First**		**Second**		**Third**		**Fourth**	
**Fifth**		**Sixth**		**Seventh**		**Eighth**	
**Ninth**		**Tenth**		**Eleventh**			

### Physical Inactivity

In 2016 a study revealed that globally, 28% of all adults aged 18 years and older were insufficiently physically active, and not following the WHO recommendation to implement at least 150 min of moderate-intensity physical activity per week (104). According to the 2019 Global Burden of Disease Study, low physical activity was ranked 18th in attributable DALYs in 2019, accounting for 198.4 age-standardized DALYs per 100,000 and 11.1 age-standardized deaths per 100,000. The EMR has the highest prevalence of insufficient physical activity than any other region. There is a clear relationship between physical inactivity and country income group globally (4). In 2016, high-income countries had more than double the prevalence of physical inactivity (37%) than low-income countries (16%), however, the situation is reversed among EMR countries where the insufficient physical activity was the highest in Kuwait followed by Saudi Arabia and UAE while the lowest was recorded in Jordan (6). According to data from the UAE national health survey 2017–2018, 70.8% of the participants did not fulfill WHO standards for adequate physical exercise. Insufficient physical activity was reported by women at a higher rate than men (74.8 and 66.8%, respectively). When compared to non-Emirates, Emiratis had a higher percentage of insufficient physical activity (80.2 and 69.2%, respectively) (105).

Physical inactivity is also a modifiable factor that is involved in upregulating the magnitude of NCDs. People who are deficiently physically active have an enhanced risk of all-cause mortality, as compared to those who perform at least 30 min of moderate-intensity physical activity on most days of the week. Additionally, physical activity lowers the risk of stroke, hypertension, and depression (106).

### Other Risk Factors Mediated by Unhealthy Diet and Obesity

#### Hypertension

Hypertension or raised blood pressure is defined as systolic and/or diastolic blood pressure greater than, or equal to, 140/90 mmHg. Hypertension is a major risk factor for heart failure, ischemic heart disease, peripheral vascular disease, renal failure, retinal hemorrhage, stroke, and dementia (107). In 2019, high blood pressure was the second-leading contributor to 235 million (95% UI 211–261) DALYs and 10.8 million (9.51–12.1) deaths in 2019 (108). Several risk factors could be involved in the upregulated blood pressure, including high salt intake, being overweight or obese, excessive use of alcohol, low or lack of physical activity, stress, air pollution, and smoking (95). Globally, in 2015, one in four men, and one in five women (i.e., 22% of the adult population aged 18 years and older) had raised blood pressure. In 2015, 28% of the population in low-income countries had high blood pressure, compared with 18% of the population in high-income countries. Reviewing the current trends demonstrated that the number of adults with high blood pressure increased from 594 million in 1975 to 1.13 billion in 2015, with the peak revealed significantly in low- and middle-income countries (109).

**Figure 6:**
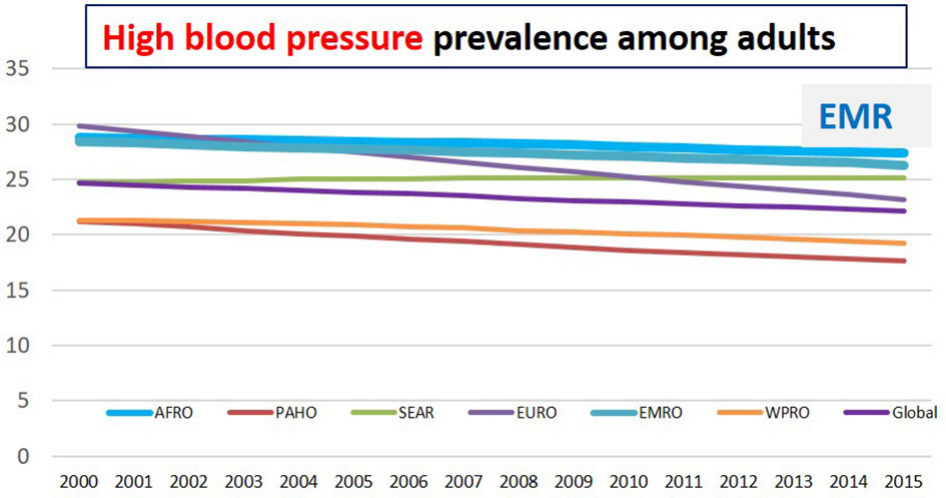
High blood pressure prevalence among adults in different regions (6).

Among all the WHO-geographical regions, EMR was the second-highest in the incidence of raised blood pressure after Africa (4). In 2015, within the EMR, the prevalence of raised blood pressure is the highest in Somalia (32.9%), then Yemen (30.7%), Afghanistan, and Pakistan (30.6% and 30.5%), respectively. In 2019, the highest prevalence of hypertension among adults was recorded in Iraq (40.7%) followed by Oman (38.6%), UAE (34.8%), Afghanistan (33.7%), Sudan (33.5%), and Kuwait (33.5%), followed by Egypt (33.2%) (6) (Table 2; Figure 6).

Raised blood pressure is the second risk factor responsible for the total number of DALYs worldwide. Among EMR countries, hypertension is the first reported risk factor responsible for the total number of DALYs in six countries (Egypt, Iran, Iraq, Morocco, Syria, and Tunisia) while it is the second reported risk factor in other nine countries (Jordan, Lebanon, Libya, Oman, Saudi Arabia, Sudan, UAE, and Yemen). The highest increase in blood pressure percentage between 2009 and 2019 in EMR was reported in UAE (140.1%) followed by Jordan (87.8%), Qatar (62.3%), Djibouti (56.9%), then Bahrain (55.7%). The increase in hypertension in the region has involved high-income countries including UAE, Qatar, and Bahrain (13) (Table 5).

#### High Fasting Blood Glucose

Accordingly, all body tissues are affected by high blood glucose including the heart, blood vessels, eyes, kidneys, and nerves, with subsequent complications including heart attack, stroke, kidney failure, lower limb amputation, blindness, and nerve damage (110). Nearly 9% of the global population had raised blood glucose levels in 2014 (111). In 2019, high fasting plasma glucose (>4.8–5.4 mmol/L) was ranked as the sixth most prevalent DALYs risk factor worldwide, accounting for 2,223.8 all-age DALYs per 100,000 and 84.0 all-age deaths per 100,000 (112). The EMR showed the highest levels (14% of the population), while 7–9% of the population from other regions had high levels of blood glucose. The upper-middle-income group tended to have higher levels (9%) (4). Within the EMR countries, the highest percentage of fasting blood glucose (≥7.0) mmol/L has been reported in Kuwait (19.6%), Qatar (18.9%), Egypt (17.9%), Saudi Arabia, Iraq (17.4%), and Jordan (16.8%) (6) (Table 2).

According to the 2019 Global Burden of Disease Study, raised fasting plasma glucose is the first reported risk factor accounted for the total number of DALYs in Palestine, while it is the second reported risk factor in three countries in the region (Bahrain, Kuwait, and Qatar) and it is the third risk factor in other seven countries (Iran, Iraq, Libya, Morocco, Oman, Saudi Arabia, and Tunisia). The highest increase in fasting plasma glucose percentage between 2009 and 2019 in EMR was reported in UAE (147%) followed by Bahrain (93.8%), Jordan and Qatar (85.9%), and Egypt (72.1%) (13) (Table 5).

#### High Cholesterol

Blood cholesterol is one of the most important risk factors for ischemic heart disease and ischemic stroke (113). The global prevalence of elevated total cholesterol (≥5 mmol/l) among adults aged ≥25 years was 38.9% (37.3% for men and 40.2% for women). Among the WHO-designated regions, the prevalence of hyper-cholesterolemia was the third highest in the EMR, at 38.4% (40.4% for women and 36.2% for men) (6). In 2018, global age-standardized mean total cholesterol was 4.6 mmol/l for women and 4.5 mmol/l for men (114) while the mean total cholesterol in the EMR is 4.4 mmol/l for both sexes, 4.4 mmol/l for men, and 4.5 mmol/l for women (6). It is noteworthy that blood non-HDL cholesterol is strongly associated with the long-term risk of atherosclerotic cardiovascular diseases. In 2018, global age-standardized mean non-HDL cholesterol was 3.3 mmol/l for women and 3.3 mmol/l (3.3–3.4) for men (114) while the mean non-HDL cholesterol in EMR in 2018, was 3.2 mmol/l for both sexes. In 2018, the highest recorded mean total cholesterol was in Lebanon 5 mmol/l followed by Kuwait and Jordan 4.8 mmol/l followed by UAE 4.6 mmol/l then Egypt and Iraq 4.4 mmol/l. Within EMR countries, the highest mean non-HDL cholesterol in 2018 was recorded in Lebanon at 3.7 mmol/l, followed by Kuwait and Jordan 3.5 mmol/l, then Oman, UAE, and Yemen 3.4 mmol/l (6) (Table 2).

According to the 2019 Global Burden of Disease Study, high LDL-c was the eighth-directing risk factor for DALYs. It contributed to 98.6 million DALYs and 4.40 million deaths in 2019 (115). High LDL-c is the fifth reported risk factor in three countries in the region (Morocco, Oman, and UAE), while it is the sixth reported risk factor in six countries in the region (Egypt, Iran, Lebanon, Libya, Syria, and Tunisia). The highest increase in fasting plasma glucose percentage between 2009 and 2019 in EMR was reported in UAE (141.5%) followed by Jordan (86.9), then Bahrain (48.6%), and Yemen (47.4%) (13) (Table 5).

### Interdisciplinary Relationships Between NCDs and Related Risk Factors

Both overweight and obesity-related to unhealthy dietary habits as well as insufficient physical activity are the key risk factors for NCDs (116). For instance, TFA consumption induces low-grade systemic inflammation and is positively correlated with endothelial dysfunction (a non-obstructive coronary artery disease) (117–121). Being overweight and having obesity also enhances low-grade systematic inflammation, creates a higher concentration of pro-inflammatory cytokines, and further endothelial dysfunction, all of which are metabolic risk factors for nutrition-related NCDs, and in particular, heart disease (122, 123) (Figure 1).

Nevertheless, its association with other risk factors, including diabetes, high body cholesterol, elevated blood pressure, and metabolic syndrome, obesity could serve as an independent risk factor for CVDs (124). Since abdominal obesity is an independent risk factor for coronary heart disease, the distribution of body fat represents an additional risk. The intra-abdominal fat buildup promotes insulin resistance, which can lead to glucose intolerance, elevated triglycerides, and low HDL as well as hypertension (125). Ultimately, obesity is the key risk factor for type 2 diabetes, cardiovascular disease, cancer, and premature death (126). Individuals who decreased 7% of their body weight significantly reduced all cardiovascular risk variables except LDL cholesterol levels, however, the rate of cardiovascular events did not decrease during the trial (127).

In a statewide cross-sectional study done by phone interviews in June 2020 in Saudi Arabia, obesity was found to be prevalent at 24.7%, and overweight at 21.7%. Type 2 diabetes, hypertension, hypercholesterolemia, sleep apnea, lung diseases, rheumatoid arthritis, colon diseases, and thyroid issues have all been significantly linked to obesity (128). A further study conducted in Qatar confirmed that obesity risk factors (c-peptide, insulin, albumin, and uric acid) and obesity-related comorbidities such as diabetes (e.g., HbA1c, glucose), liver function (e.g., alkaline phosphatase, gamma-glutamyl transferase), lipid profile (e.g., triglyceride, LDL-c, HDL-c), as well as most of the dual-energy x-ray absorptiometry measurements (e.g., bone area, bone mineral composition, bone mineral density, etc.) were significantly (*p* <0.05) higher in the obese group (129).

Substantially, elevated blood pressure has been linked to the consumption of food high in salt and NCDs. An intervention trial that included 9,000 adults with baseline systolic blood pressure between 130 and 180 mmHg indicated that a lower blood pressure target was accompanied by a significantly lower incidence of myocardial infarction, acute coronary syndrome, stroke, heart failure, or death (130). Diabetes also is a recognized and significant risk factor for CVDs (131). CVDs is the leading cause of morbidity and mortality among individuals with diabetes. It is therefore recommended that individuals with diabetes should have a target blood pressure of <130/80 mmHg to prevent the incidence of CVDs (132).

## Discussing the Cultural and Sociodemographic Effect on the NCDs Related Risk Factors

Of the 22 countries and territories in the EMR, 16 are considered low-income or middle-income countries. Several countries in the EMR have lengthy histories of political instability, war, and social conflict, which have resulted in the large-scale internal and external displacement of citizens; half of the region's countries and territories are now under an acute or chronic state of emergency. These socioeconomic determinants of health, as well as accompanying inequities, have an impact on health status and access to care throughout the EMR as well as access to healthy food, which is unsurprising. Moreover, disease epidemiological data on disease incidence, prevalence, and management are scarce and lacking (133). Furthermore, NCDs mortality, and its social, environmental, behavioral, nutritional, and clinical determinants are not distributed evenly within countries (134). The most deprived communities have a higher risk of premature death than those in the most affluent. Therefore, reducing national-level NCDs risk requires actions that address the disproportionate burden in deprived communities (29).

The lowest risk of NCDs mortality is seen in high-income countries in western Europe, Asia-Pacific, Australia, and Canada, whereas, the highest risk was observed in low-income and middle-income countries. The highest probabilities were seen in parts of sub-Saharan Africa, and Guyana. In EMR, Yemen, and Afghanistan (one in four to one in three people are at risk of dying from NCDs), people are about 3–7 times more likely to die than those in high-income countries. Similarly, the probability of dying from NCDs between the age of 30 and 70 in EMR is 24.5% (one in four adults will die before the age of 70) (29).

Literature on this subject usually shows a positive association between socioeconomic status and obesity in low-income countries. However, contrary to this, a multinomial regression analysis study conducted in Cairo, Egypt reported no significant associations between most SES spectrum and overweight/obesity in the studied population. The study suggested that obesity programs and policies should be targeted at all socioeconomic status groups in Egypt (135). A study conducted in Jordan revealed that the prevalence of overweight/obese women was 70.6%. Furthermore, the association between age and overweight/obesity was significant (*p* < 0.0001). The high prevalence of overweight/obesity among women in Jordan was related to high parity and low education level (136). Conversely, in research conducted in Saudi Arabia, the prevalence of overweight and obesity in men was 35.1% and 34.8%, respectively, and in women, it was 30.1% and 35.6%. Obesity and overweight increased in prevalence until 60 years of age, then declined in both sexes in the oldest age group. After adjusting for age, earning a postgraduate degree raised the risk of obesity in men, but increased physical activity decreased it in both sexes. Obese women had a higher risk of prediabetes and diabetes, obese males had a higher risk of hypertension, and both sexes had a higher risk of dyslipidemia. A familial history of dyslipidemia was linked to a lower risk of obesity in women, whereas women who were overweight were more liable to develop prediabetes, diabetes, and dyslipidemia, while men who were overweight were more liable to hypertension (137).

Analyzing the data from a population-based cross-sectional survey of diabetes and obesity in Kuwait, revealed that the prevalence of overweight, obesity, and central obesity were 40.6%, 42.1%, and 73.7%, respectively. Men were 26% more likely than women to be overweight, while women had 54% and seven-fold higher probabilities of obesity and central obesity, respectively. Young adults aged 18–29 years have a significant prevalence of obesity and overweight. Obesity/central obesity was associated with higher educational attainment, physical activity, and being non-Kuwaiti. Smoking history, high blood pressure, higher income, and marital status are all linked to an increased risk of obesity/central obesity (138). In another cross-sectional study conducted among 3,915 Kuwaiti adults, obesity prevalence was 40.3% (men, 36.5%; women, 44.0%); and overweight prevalence was 37% (men, 42%; women, 32.1%). Obesity prevalence was linked to female sex, age, diabetes history, and marital status in both men and women, but was inversely linked to education level in women. Men were more likely to have an increased waist-to-hip ratio (46.91%) as compared to women (37.9%). In both men and women, waist circumference, waist-hip, and waist-height ratios were found to be directly associated with diabetes and negatively associated with education level in women (139).

In a study conducted in Libya that explored the key risk and protective factors beyond the high prevalence rates of overweight and obesity, 11 factors were identified to be associated with obesity among men and women. These include socio-demographic and biological factors, socioeconomic status, unhealthy eating behaviors, knowledge about obesity, social-cultural influences, healthcare facilities, physical activity, the effect of the neighborhood environment, sedentary behavior, food-subsidy policy, and suggestions for preventing and controlling obesity (140). Another cross-sectional survey revealed that the prevalence of obesity, overweight, and normal weight among Libyan adults was 42.4%, 32.9%, and 24.7%, respectively. Women were more likely than men to be overweight or obese (the prevalence of overweight was 33.2% in women vs. 32.4% in men, and the prevalence of obesity was 47.4% in women versus 33.8% in men) (141).

## Sustainable Development Goals Target 3.4: Pathways and Forward Steps

The Sustainable Development Goals (SDGs) target 3.4 is to reduce NCDs-related premature mortality by a third by 2030 compared to 2015 levels, as well as to enhance mental health and wellbeing through prevention and treatment (142). It has been reported that the progress in most international countries is too slow to meet this goal (143).

Although SDG target 3.4 is the same, differences exist between countries in terms of risk of dying from various NCDs (29, 144). Throughout this review, the percentages of different risk factors associated with NCDs incidence have been elaborated. This is important to highlight the pathways through which each country can achieve SDG target 3.4 and to guide governments and donors in prioritizing resources and interventions in their national NCDs response.

Based on 2010–2016 trends, women in 17 of 176 (9.7%) countries and men in 15 of 176 (8.5%) countries are expected to achieve SDG target 3.4 by 2030. The high-income countries that are on track include Denmark, Luxembourg, New Zealand, Norway, Singapore, and South Korea as well as central and eastern European countries. Furthermore, NCDs death rates among men and women in EMR countries as Iran are falling quickly enough to meet the 2030 target. Kuwaiti women and Bahraini men are likewise on pace (29). In contrast, the risk of dying from NCDs is expected to remain stable or increased among women in 14 (8%) countries and men in 20 (11.4%) countries according to 2010 and 2016 trends. Bangladesh (men), Egypt (women) from EMR, Ghana (men and women), Côte d'Ivoire (men and women), Kenya (men and women), Mexico (men), Sri Lanka (women), Tanzania (men), and the United States (women) were involved. This could be referred to the changes in population size and age structure, even if the risk of dying from NCDs reduces, the number of deaths from NCDs may continue to rise (29).

According to a new World Health Organization report, if low and lower-middle income nations invest less than a dollar per person per year in the prevention and treatment of NCDs, close to seven million deaths could be avoided by 2030 (145)^1^. These include low-cost strategies for reducing tobacco and alcohol use, improving diets, increasing physical activity, lowering the risk of cardiovascular disease and diabetes, and preventing cervical cancer (145) (see text footnote ^1^).

The regional framework for action on obesity prevention 2019–2023 (146), set a road map for countries of the region to accelerate the action on NCDs and obesity prevention. It sets out six key action areas for improving nutrition and food security including, sustainable, resilient food systems for healthy diets; aligned health systems providing universal coverage of essential nutrition actions; social protection and nutrition education; trade and investment for improved nutrition; safe and supportive environm ents for nutrition at all ages; and strengthened governance and accountability for nutrition (17, 146)^2^.

By investing in the Best Buy policies, countries will protect people from NCDs. Best Buy actions include increasing health taxes, restrictions on marketing and sales of unhealthy dietary products, food labeling, and education. They also include actions connected to managing metabolic risk factors, such as hypertension and diabetes, to prevent more severe disease or complications (145) (see text footnote ^1^). Table 6 reveals the key policies and action plans available and implemented among EMR countries (6, 17–20).

**Table 6:** The polices available and implemented in EMR countries.

	**Development of national nutrition strategy or action plan**	**Plan of action for obesity prevention**	**Front-of-pack nutrition labeling**	**Policy to reduce salt/sodium consumption**	**Policy to limit trans-fatty acids intake**	**Policy to reduce the impact of marketing of food to children**	**Tax on sugar sweetened beverages**	**Food-based dietary guidelines**
Afghanistan	3 (16)	✘ (16)	✘ (19)	✘ (6)	✘ (6)	✘ (6)	✘ (6, 17)	3 (16)
Bahrain	3 (16)	3 (16)	✘ (18)	3 (6, 16, 19)	3 (6, 16, 20)	3 (6, 16)	3 (6, 17, 147)	3 (16)
Djibouti	3 (148)	✘ (16)	✘ (19)	✘ (6)	✘ (6)	✘ (6)	✘ (6, 17)	✘ (16)
Egypt	3 (16, 149)*	3 (16)	✘ (19)	3 (6, 16, 19)	3 (20)	3 (16)*	3 (149)	✘ (16)
Iran	3 (16)	3 (16)	3 (16, 18, 19)	3 (6, 16, 19)	3 (6, 16, 20)	3 (6, 16)	3 (6, 17)	3 (16)
Iraq	3 (16)	✘ (16)	✘ (19)	3 (6, 17)	3 (20)	✘ (6)	✘ (6, 17)	✘ (16)
Jordan	3 (16, 149)*	3 (16)	✘ (19)	3 (6, 16, 17, 19)	3 (6, 16, 20)	3 (16)*	✘ (6, 17)	3 (16)
Kuwait	3 (16)	3 (16)	✘ (19)	3 (6, 16, 19)	3 (6, 16, 20)	3 (16)*	3 (147)	✘ (16)
Lebanon	3 (16)	3 (16)	✘ (19)	✘ (6)	✘ (6)	3 (16)*	✘ (6, 17)	3 (16)
Libya	✘ (16)	3 (16)	✘ (19)	✘ (6)	✘ (6)	✘ (6)	✘ (6, 17)	3 (149)
Morocco	3 (16)	3 (16)	3 (16, 18, 19)	3 (6)	3 (6, 20)	3 (6, 16)*	3 (6, 17)	3 (16)
Oman	3 (16)	3 (16)	✘ (19)	3 (6, 16, 19)	3 (6, 16, 20)	3 (6, 16)*	3 (6, 17, 147)	3 (16)
Pakistan	3 (16)	✘ (16)	✘ (19)	✘ (19)	3 (20)	3 (149)	✘ (6, 17)	✘ (16)
Palestine	3 (149)	3 (149)*	✘ (19)	3 (17, 19)	3 (20)	✘ (6)	3 (149)	3 (149)*
Qatar	3 (16)	3 (16)	✘ (19)	3 (6, 19)	3 (6, 16, 20)	3 (16)	3 (17, 147)	3 (16)
Saudi Arabia	3 (16)	3 (16)	3 (16, 18, 19)	3 (6, 16, 19)	3 (6, 16, 20)	3 (16)	3 (6, 17, 147)	3 (16)
Somalia	3 (16)	✘ (16)	✘ (19)	✘ (6)	✘ (6)	✘ (6)	✘ (6, 17)	✘ (16)
Sudan	3 (16)	3 (16)	✘ (19)	✘ (6)	✘ (6)	✘ (6)	✘ (6, 17)	3 (16)
Syria	3 (16)	3 (16)	✘ (19)	✘ (6)	✘ (20)	✘ (6)	✘ (149)	3 (16)
Tunisia	3 (16)	3 (16)	3 (16, 18, 19)	3 (6, 19)	3 (6, 20)	✘ (6)	3 (6, 17)	✘ (16)
UAE	3 (16)	✘ (16)	3 (16, 18, 19)	3 (6, 19)	3 (6, 20)	3 (149)*	3 (6, 17, 147)	3 (16)
Yemen	3 (16)	3 (16)	✘ (19)	✘ (6)	✘ (2)	✘ (6)	✘ (6)	✘ (16)

The interventions have already been used successfully in many countries around the world. Among EMR countries that are on track to meet SDG target 3.4 are Iran, Kuwait, and Bahrain (29). These three countries have policies to reduce salt/sodium consumption, tax on sugar sweetened beverages, policy to eliminate industrially produced trans-fatty acids, policy to limit saturated/ trans-fatty acids intake, policy to reduce the impact of marketing of food to children, and policy on salt iodization (6, 17–20) (see Table 6).

## Conclusion

Among the top causes of morbidity and mortality related to nutrition in EMR are cardiovascular heart diseases followed by cancer and then diabetes. Globally, the disease burden attributable to hypertension, alcohol consumption, high body mass index, high fasting blood glucose, high sodium intake, and unhealthy diet consumption is increasing significantly, while the disease burden attributable to children being underweight, suboptimal breastfeeding, and micronutrient deficiencies have all decreased significantly. Among the EMR countries, UAE followed by Jordan revealed a significant increase in the percentage change of nearly all the risk factors that are involved in NCDs causing morbidity and mortality (150).

The data and correlation figures included in this study represent evidence that constitutes a significant public health concern about the relationship between unhealthy diet consumption and obesity that further induces other risk factors including (hypertension, insulin resistance, and a systemic inflammatory milieu), leading to NCDs (Figure 1). It is therefore important to recognize the key therapeutic modalities for treating and prohibiting NCDs, which are to fight against weight gain and obesity and to advocate lifestyle-based therapies; including proper nutrition and regular physical activity. These are the key therapeutic modalities that will reduce the risk of NCDs. Additionally, body mass index should be used as a first step in establishing the criteria to judge potential health risks.

Countries in the EMR need to continue building on the achieved progress and scale up action across the region while boosting efforts in areas where concrete action is absent through the following key stakeholders to reach the agreed global and regional goals relating to nutrition and diet-related NCDs. This could be achieved through the following key stakeholders, Governments can provide and improve access to quality NCDs and obesity care, as well as develop and implement policies that promote and normalize healthy eating and living, in addition to banning the marketing of unhealthy foods and beverages high in fat, sugar, and salt. Civil society groups, including non-governmental organizations and the media, can work with individuals and communities to educate and diffuse key messages on the root causes of NCDs and obesity, the importance of prevention and treatment, as well as the impact of adopting healthy behaviors like keeping physically active and choosing healthy food and drinks. Health care professionals, whether working directly in NCDs and obesity care or supporting and working with those living with obesity, can learn more about obesity, expand their knowledge, and have up-to-date, evidence-based obesity management resources to help them understand and address the root causes of this disease. Individuals and families can adopt healthier behaviors, share experiences, as well as ask for support, whilst also supporting others to improve their health and well-being and that of their children (17) (see text footnote ^2^).

Countries in EMR are encouraged to adopt and implement the regional nutrition strategy for nutrition 2020–2030 (21), the regional framework for action on obesity prevention 2019–2023 (151), and the regional framework for action to implement the United Nations Political Declaration on the NCDs (152).

## Author Contributions

All authors listed have made a substantial, direct, and intellectual contribution to the work and approved it for publication.

## Conflict of Interest

The authors declare that the research was conducted in the absence of any commercial or financial relationships that could be construed as a potential conflict of interest.

## Publisher's Note

All claims expressed in this article are solely those of the authors and do not necessarily represent those of their affiliated organizations, or those of the publisher, the editors and the reviewers. Any product that may be evaluated in this article, or claim that may be made by its manufacturer, is not guaranteed or endorsed by the publisher.

## Footnotes

^1^Available online at: https://www.who.int/news/item/13-12-2021-investing-1-dollar-per-person-per-year-could-save-7-million-lives-in-low-and-lower-middle-income-countries.

^2^Available online at: https://apps.who.int/iris/bitstream/handle/10665/346443/EMRC68INFDOC8-eng.pdf.
